# Electroacupuncture alleviates pain by activating the MD2/TLR4/NF-κB pathway in the ST36 acupoint

**DOI:** 10.3389/fimmu.2025.1626755

**Published:** 2026-01-29

**Authors:** Jiangjiang Fu, Gangchen Cheng, Zhongxi Lyu, Zezhi Fan, Feiyang Li, Zhifang Xu, Bo Chen, Yongming Guo, Yuhan Liu, Hui Zhang, Yuan Xu, Yi Guo

**Affiliations:** 1 School of Acupuncture & Moxibustion and Tuina, Tianjin University of Traditional Chinese Medicine, Tianjin, China; 2 Research Center of Experimental Acupuncture Science, Tianjin University of Traditional Chinese Medicine, Tianjin, China; 3 National Clinical Research Center for Chinese Medicine, Tianjin, China

**Keywords:** electroacupuncture, analgesia, ST36 acupoint, MD2/TLR4/NF-κB pathway, Grem1/BMP4/COX2 pathway

## Abstract

**Introduction:**

The acupuncture acupoint is the critical initial site for the therapeutic efficacy of electroacupuncture (EA). Previous studies have confirmed that the NF-κB pathway within the acupoint region mediates the therapeutic effects of acupuncture. Therefore, this study focuses on an in-depth investigation of the MD2/TLR4/NF-κB axis.

**Methods:**

The mouse model of adjuvant-induced arthritis (AIA) was established via intraplantar injection of Complete Freund’s Adjuvant (CFA). EA intervention was applied bilaterally to the Zusanli (ST36) acupoints, and behavioral, molecular, and immunological approaches were integrated to investigate the role of MD2 in EA-mediated analgesia.

**Results:**

EA increased paw thermal withdrawal thresholds (PTWTs) in AIA mice (*P* < 0.05), accompanied by higher levels of MD2, TLR4, p65, and the phosphorylated form of p65 (p-p65) at the acupoint. Co-immunoprecipitation (Co-IP) confirmed binding between MD2 and TLR4 in ST36 acupoint, while immunofluorescence (IF) revealed co-localization of TLR4 with fibroblasts and mast cells in ST36, suggesting these immune cells are critical targets for signal activation. Lentivirus-mediated knockdown of MD2 in the acupoint partially reversed EA’s analgesic effects and suppressed downstream TLR4/NF-κB pathway activation, whereas MD2 overexpression elicited a partial analgesic effect and promoted pathway activation. Together with spinal cord proteomics data, these findings indicate that modulating MD2 in the acupoint can regulate spinal cord-related signaling pathways. Mechanistically, EA dynamically regulates the equilibrium of the Grem1/BMP4/COX2 axis in the spinal dorsal horn via activation of the MD2/TLR4/NF-κB pathway cascade, achieving systemic analgesia.

**Conclusion:**

This study provides molecular evidence supporting the "acupoint priming" theory in acupuncture and highlights MD2 as a potential therapeutic target for pain management.

## 1 Introduction

Pain is one of the most common symptoms in clinical medicine, severely affecting patients’ quality of life and mental health. With the continuous development of modern medicine, pain relief methods have become increasingly diversified, ranging from pharmacological treatments to physical therapies and psychological interventions. However, these approaches still face significant challenges, such as limited analgesic efficacy and notable side effects. In this context, EA, which integrates traditional acupuncture with modern electrical stimulation techniques, has emerged as an innovative therapy with certain advantages in pain relief. Randomized controlled trials have already demonstrated the effectiveness of EA in alleviating pain (1–4).

In recent years, research on EA has made significant progress in the field of pain relief. The analgesic effects of EA involve the interactions of multiple systems from the periphery to the central nervous system. The analgesic signals of EA originate from the local acupoints, through the spinal cord, and to the brain, undergoing multiple stages and involving the nervous, immune, and endocrine systems (5–9). The mechanisms explored in related studies include: at the local acupoints, EA can modulate peripheral inflammatory mediators, neurotransmitters, and nociceptive ion channels. At the spinal cord level, EA promotes the release of endogenous analgesic substances (e.g., opioid peptides and endocannabinoids), downregulates the release of glutamate, upregulates the release of neurotransmitters in the descending pain modulatory system, inhibits ascending excitatory pathways, attenuates central sensitization, and regulates neuroplasticity. At the higher brain level, EA increases connectivity within the default mode network, sensorimotor network, and brain regions related to pain, emotion, and memory, regulates cerebral blood flow, and induces neuroregeneration. Although current studies have partially elucidated the mechanisms from diverse perspectives, research on localized acupuncture domains remains insufficient, especially in addressing methodological challenges such as standardized effect evaluation, data acquisition limitations, and the integration of emerging technologies.

Acupoints are composed of the epidermis, dermis, subcutaneous tissue, muscle, and related structures (such as nerves, blood vessels, lymphatic vessels, and tendons), and serve as the primary sites for EA. Some studies have found that acupuncture can increase the number of fibroblasts in the dermis and connective tissue layers of the skin, prompting them to release a variety of chemical substances, such as IL-1α, IL-1β, IL-6, IL-7, IL-18, TNF-α (10). These chemical substances can further act adjacent nerve endings and cells, thereby initiating the transduction of acupuncture signals (11, 12). Similarly, mast cells at the acupoint play important roles in the analgesic effect of acupuncture. Some researchers have found that the analgesic effect of acupuncture is significantly weakened after the injection of sodium cromoglycate (a mast cell stabilizer) to inhibit mast cells at the acupoint (13).

Myeloid differentiation protein 2 (MD2), a secretory glycoprotein composed of 160 amino acids with two relatively independent functional domains, is a crucial accessory factor in the Toll-like receptor 4 (TLR4) signaling pathway and plays a significant role in pain responses (14). Research has shown that in a chronic pain model, MD2 expression is upregulated in neurons of the spinal dorsal horn in mice, and EA treatment can reduce MD2 expression and reverse pain behaviors (15). Studies have found that MD2 can combine with TLR4 intracellularly for co-secretion or be secreted alone extracellularly, primarily by immune cells (e.g., macrophages) and fibroblasts (16, 17). Its extracellular region, rich in leucine-rich repeats (LRRs), not only associates with TLR4 but also exists in a soluble form in the blood, playing an essential role in the translocation and membrane expression of TLR4 (18–20). The TLR4 system serves as a central hub of the innate immune system, functioning as a highly complex and precisely regulated signaling network that exhibits a characteristic dual nature: appropriate activation promotes host defense, whereas dysregulated activation transforms it into a pathogenic driver of disease (15). More importantly, MD2 can promote the synthesis and release of inflammatory factors by activating the TLR4 and NF-κB pathway in fibroblasts (17), which is notably consistent with our research findings. However, the mechanisms by which MD2 participates in acupoint-mediated analgesia remain unclear. This study aims to systematically investigate the role of the MD2/TLR4 pathway within acupoints during EA intervention.

Based on existing research, we hypothesize that EA stimulation of the ST36 acupoint induces MD2 release in acupoint tissue, which activates TLR4 on key cells (e.g., fibroblasts, mast cells, macrophages) in the inflamed skin. This activation continuously stimulates the NF-κB pathway, which we assess by the NF-κB pathway, promoting the synthesis and release of inflammatory factors in the acupoint area. These factors activate peripheral nerves to transmit acupuncture signals, these peripheral signals converge at the spinal cord level, where they regulate the balance of analgesia-related neurotransmitters or modulators within the dorsal horn, ultimately producing a cumulative analgesic effect.

## 2 Materials and methods

### 2.1 Experimental animal and ethics statement

We purchased 8-week-old, specific-pathogen-free (SPF) level, inbred male C57BL/6J mice, each weighing 20g ± 2g, from the Experimental Animal Science Department of the Beijing Vital River Laboratory Animal Technology Co., Ltd. The Animal Qualification Certificate No. is SCXK [Beijing] 2021 - 0006, and the Laboratory Use License No. is SYXK (Jin) 2020 - 0005. All animal experiments were approved by the Tianjin University of Traditional Chinese Medicine Committee on Laboratory Animals (protocol number: TCM-LAEC2022232) and conducted in accordance with the latest NIH guidelines for the Care and Use of Laboratory Animals. All mice were housed in a temperature - controlled environment with a 12-hour light-dark cycle and had free access to food and water in their cages.

The experimental timeline was defined relative to the day of model induction (designated as Day 0). The EA intervention period spanned from Day 1 to Day 7 (D1-D7). Key timepoints were designated as follows: the lentivirus injection was administered on D3; the period from D-3 to D-1 was allocated for animal acclimation; model establishment was confirmed on D1 pre (20 hours post-model induction) via behavioral testing; post-intervention behavioral assessments and tissue collection were performed on D1 post (30 minutes after EA intervention) and subsequently on D3, D5, and D7.

### 2.2 Inflammatory pain model

**Figure 1:**
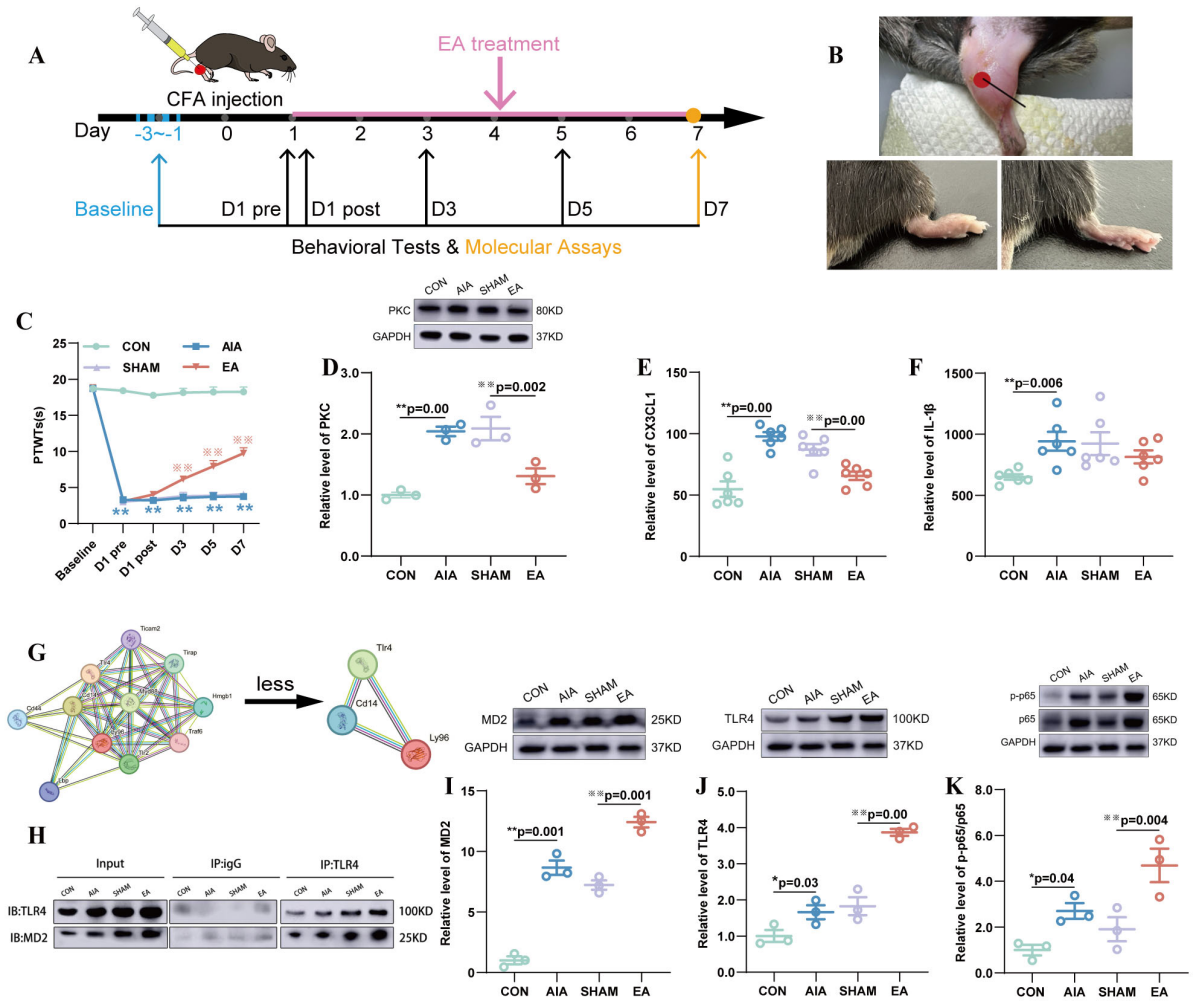
EA-induced analgesia is associated with MD2, TLR4, and p-p65/p65 in the ST36 acupoint and MD2 and TLR4 interactions. **(A)** The experimental protocol timeline is illustrated, with the duration of each intervention clearly indicated in the figure. **(B)** The location of the ST36 acupoint in mice (up) and the swelling of paw comparison before (right) and after (left) CFA injection. **(C)** PTWTs were detected on the D-3 to D-1, D1pre, D1post, D3, D5, and D7 after interventions (n = 8), ^**^P < 0.01 vs. the CON, ^※※^*P* < 0.01 vs. the SHAM. **(D)** The expression levels of PKC protein in the spinal cord were determined by WB (n = 3), ^**^P < 0.01 vs. the CON, ^**^P < 0.01 vs. the SHAM. **(E, F)** The expression levels of CX3CL1 **(E)**, IL-1β **(F)** in the spinal cord were determined by Elisa (n = 6), ^**^P < 0.01 vs. the CON, ^**^P < 0.01 vs. the SHAM. **(G)** Data sourced from *https://cn.string-db.org/*. **(H)** Co-IP assay with TLR4 antibody (or IgG) in the ST36 acupoint, followed by WB analysis of MD2 and TLR4. **(I–K)** The expression levels of MD2 **(I)**, TLR4 **(J)**, and p-p65/p65 **(K)** were determined using WB analysis in the ST36 acupoint on D7 (n = 3), ^*^P < 0.05 vs. the CON, ^**^P < 0.01 vs. the CON, ^※※^*P* < 0.01 vs. the SHAM.

The AIA model was established by injecting 50 µL of CFA (1 mg/mL, Sigma, St. Louis, MO, USA) into the right hindlimb footpads of mice. After 24 hours, the development of abnormal redness and swelling in the right foot (**Figure 1B**), decreased movement, limping, and a significant decrease in PTWTs indicated successful modeling. Control mice were intradermally injected with 50 µL of normal saline (0.9%).

**Figure 2:**
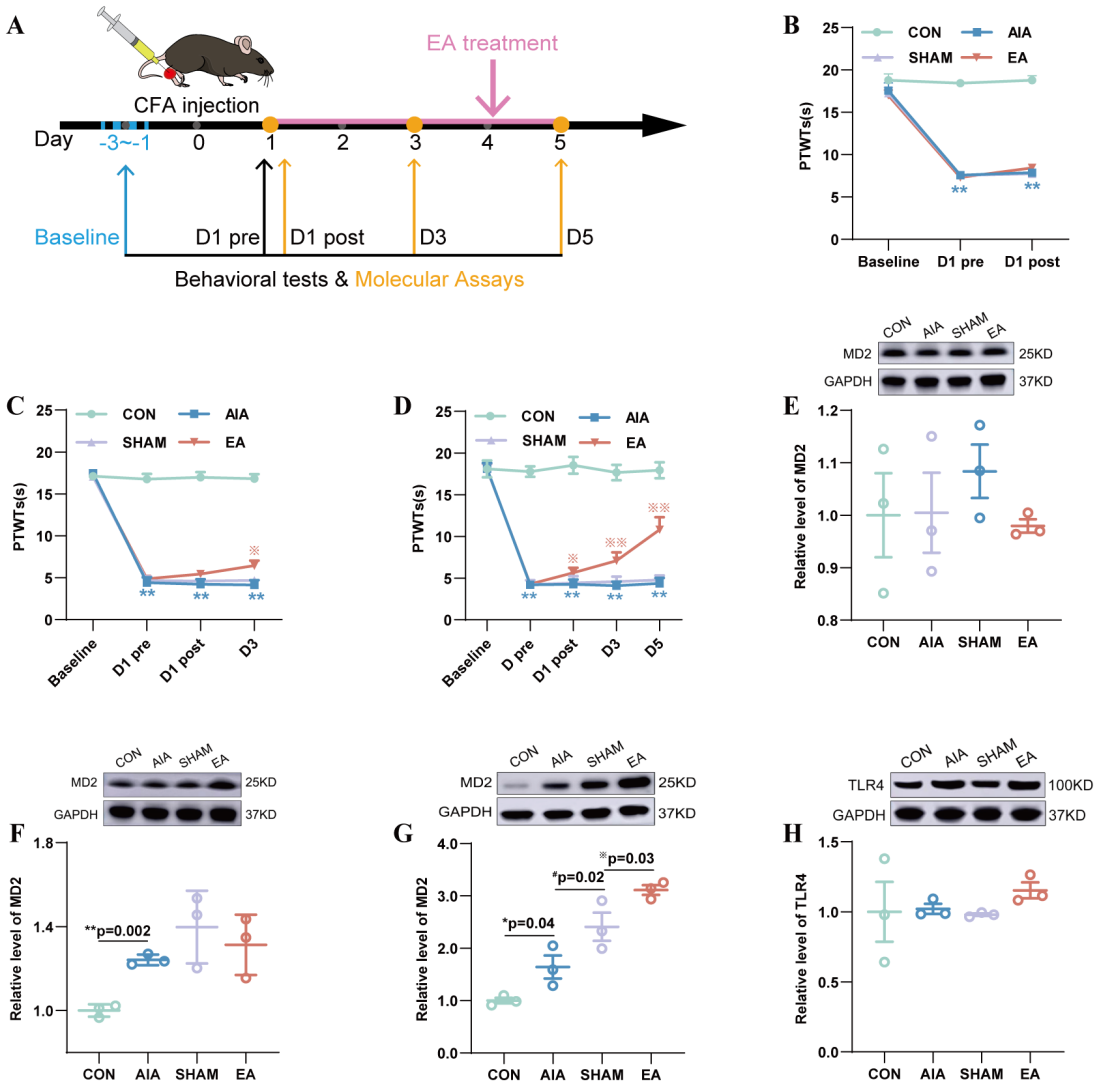
The pattern of the effect of EA treatment on MD2, TLR4 in ST36 acupoint. **(A)** Behavioral testing, acupoint stimulation and molecular detection experimental schedule. **(B–D)** PTWTs were detected on D1post **(B)** (n=8), D3 **(C)** (n=6), and D5 **(D)** (n=6), ^**^P < 0.01 vs. the CON, ^※^*P* < 0.05 vs. the SHAM, ^※※^*P* < 0.01 vs. the SHAM. **(E–H)** The expression level of MD2 in the ST36 acupoint was determined by WB on D1post **(E)**, D3 **(F)** and D5 **(G)** (n = 3); the TLR4 was determined on the D5 **(H)** (n = 3), ^*^P < 0.05 vs. the CON, ^**^P < 0.01 vs. the CON, #*P* < 0.05 vs. the AIA. ^※^*P* < 0.05 vs. the SHAM.

### 2.3 Intervention measures

For EA treatment at the bilateral ST36 acupoints, needles (0.16 mm × 7 mm) were inserted into regions located about 3–4 mm down from the knee joint and 2–3 mm lateral to the anterior tubercle of the tibia. Following acupuncture insertion, subtle rhythmic contractions were observed in the local muscles. Subsequently, the needle handles were connected to the positive pole of the EA device (SEMZ-V, Hua Tuo, Shanghai Huanxi Medical Device Co., Ltd., China), the negative pole was connected to a cotton thread (pre-soaked in physiological saline solution to ensure good conductivity) wrapped around the mouse’s ankle. The stimulation parameters were set as an alternating dense-sparse frequency mode of 2/15 Hz, with a current intensity of ≤0.05 mA, for a duration of 30 minutes. Mice in the SHAM group underwent identical needle insertion and device connection but the current output was adjusted to 0 mA for 30 minutes. Mice in the CON and AIA groups were restrained for the same duration but did not receive any needle insertion or treatment.

### 2.4 Study procedure

This experiment was divided into three parts, with the detailed research procedures as follows:

Experiment 1: The mice were randomly assigned to 4 groups: control (CON) group, model (AIA) group, the sham EA treatment (SHAM), EA treatment (EA) group. The duration of the experiment was 11 days. The mice received the PTWTs testing as the baseline values on D-3 to D-1. On D0, the mice were grouped based on the baseline values and then subjected to modeling. On D1pre (24 hours after model establishment, 30 minutes before treatment) all mice received the PTWTs testing to assess whether the modeling was successful. The PTWTs testing was carried out on D1post to D7 (every other day, 30 minutes after treatment) to evaluate the analgesic effects of EA. Intervention (sham EA or EA treatment) were applied to mice in the SHAM groups and EA groups on D1post to D7 (once a day). To detect cytokine levels and perform immunofluorescence localization analysis, tissues from the ST36 acupoint were collected after behavioral tests and interventions on D7.

Experiment 2: The procedures for grouping, modeling, and intervention in this experiment are the same as those in Experiment 1. To observe the changes in the expression levels of MD2, TLR4 and p-p65/p65 at the specific time, the ST36 tissue samples were collected and tested after the PTWTs were measured on D1post, D3, D5 and D7.

Experiment 3: To investigate whether MD2 in the ST36 acupoint mediated the analgesic effects of EA by activating the TLR4 and NF-κB pathway, we used lentivirus to specifically regulate the expression of MD2 in the ST36 acupoint. This experiment was divided into two parts:

In the first part, knockdown lentivirus was injected into the ST36 acupoint. Mice were divided into three groups: AIA+LVcon (model mice injected with control lentivirus), AIA+LVcon+EA (model mice injected with control lentivirus and treated with EA), and AIA+LV+EA (model mice injected with knockdown lentivirus and treated with EA). The modeling, and intervention procedures were identical to those in Experiment 1. Lentivirus injection was performed 4 days prior to EA intervention and repeated on D5. On D7, mice were euthanized, and tissues from the acupoint and spinal cord (L3-L5) were collected. These tissues were then analyzed by proteomic analysis and WB.

In the second part, overexpression lentivirus was injected into the ST36 acupoint. Mice were divided into four groups: AIA+OEcon (model mice injected with control overexpression lentivirus), AIA+OE (model mice injected with overexpression lentivirus), AIA+OEcon+EA (model mice injected with control overexpression lentivirus and treated with EA). Lentivirus injection was performed 4 days prior to EA intervention. On Day 7, tissues from the ST36 acupoint were collected and analyzed using WB.

### 2.5 Behavioral assessment

The BME-410C thermal radiation pain instrument (Chinese Academy of Medical Sciences Institute of Biomedical Engineering, China) was used to measure the PTWTs on the modeled side of the mouse. Prior to testing, mice were acclimatized to the environment of a plastic box with a glass plate for 30 minutes. The laboratory temperature was maintained at 23-25°C, with humidity controlled at 40-60%. After the mice had calmed down, a radiation source was applied to the plantar surface of the right hind paw from beneath the glass floor, and paw withdrawal latency was measured and recorded. PTWTs were measured once every 5 minutes, for a total of three measurements, with the average value recorded. To prevent scalding, the upper limit of the thermal pain threshold was set at 30 seconds. The behavioral test was conducted by two observers, using a blinded method for the experimental group.

### 2.6 Western blotting

These tissues were lysed in a mixture of RIPA buffer (R0010, Solarb, China), PMSF (P0100, Solarb, China), and protein phosphatase inhibitor (P1260, Solarb, China). The total protein concentration was subsequently determined using a BCA protein assay kit (AR0146, BOSTER, China). The samples were then denatured by adding sample buffer and boiling at 100°C for 10 minutes. Next, 30 μg protein was separated on 10% SDS-PAGE gels in Tris-glycine buffer containing 0.1% SDS and was transferred to polyvinylidene fluoride (PVDF) membranes in a transfer buffer composed of 25 mM Tris, 192 mM glycine, and 20% methanol at 100 V for 95 minutes. The membrane was then incubated in 5% skim milk at room temperature for 2 hours to block non-specific binding. The blots were then washed and incubated overnight at 4°C with anti-MD2 antibody (1:1000, sc-80183, Santa, USA; 1:1000, ab24182, abcam, UK), anti-TLR4 antibody (1:1000, sc-293072, Santa, USA; 1:1000, 19811-1-AP, Proteintech Group, China), anti-P-P65 antibody (1:1000, 3033S, Cell Signaling Technology, USA), anti-P65 antibody (1:8000, 8242, Cell Signaling Technology, USA), anti-Grem1 antibody (1:800, sc-293426, Santa, USA), anti-BMP4 antibody (1:200, 12492-1-AP, Proteintech Group, Inc, USA), anti-COX2 antibody (1:500, A3560, ABclonal, China), anti-GAPDH antibody (1:3000000, A19056, ABclonal, China). Subsequently, the membranes were washed and incubated with the corresponding secondary antibody for 2 hours at room temperature. Protein-antibody complexes were visualized using an electro-generated chemiluminescence (ECL) detection system. GAPDH was used as an internal control.

**Figure 3:**
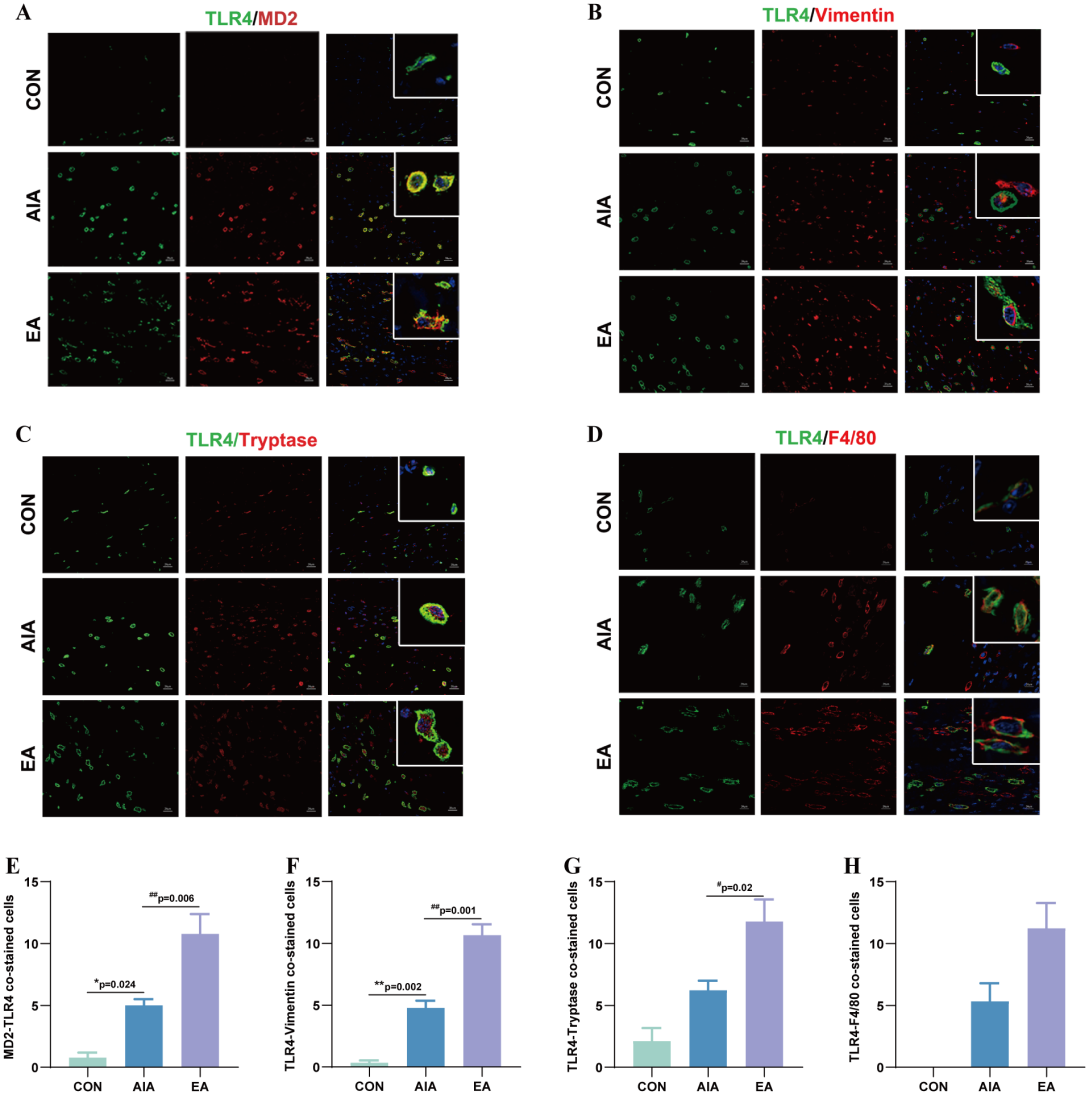
EA treatment can increase the co-expression of MD2/TLR4, TLR4-Vimentin, TLR4-Tryptase, as well as TLR4-F4/80 at the ST36 acupoint. **(A)** Double-labeling immunofluorescence of TLR4 (green), MD2 (red) and DAPI (blue), scale bar = 20 µm. **(B)** Double-labeling immunofluorescence of TLR4 (green), fibroblasts (red) and DAPI (blue), scale bar = 20 µm. **(C)** Double-labeling immunofluorescence of TLR4 (green) and Tryptase (red), scale bar = 20µm. **(D)** Double-labeling immunofluorescence of TLR4 (green) and F4/80 (red), DAPI (blue), scale bar = 20µm. **(E)** Immunofluorescence quantitative analysis of MD2/TLR4 (n = 3), ^*^P < 0.05 vs. the CON, ^##^*P* < 0.01 vs. the AIA. **(F)** Immunofluorescence quantitative analysis of TLR4-Vimentin (n = 3), ^**^P < 0.01 vs. the CON, ^##^*P* < 0.01 vs. the AIA. **(G)** Immunofluorescence quantitative analysis of TLR4-Tryptase (n = 3), ^#^*P* < 0.05 vs. the AIA. **(H)** Immunofluorescence quantitative analysis of TLR4-F4/80 (n = 3).

**Figure 4:**
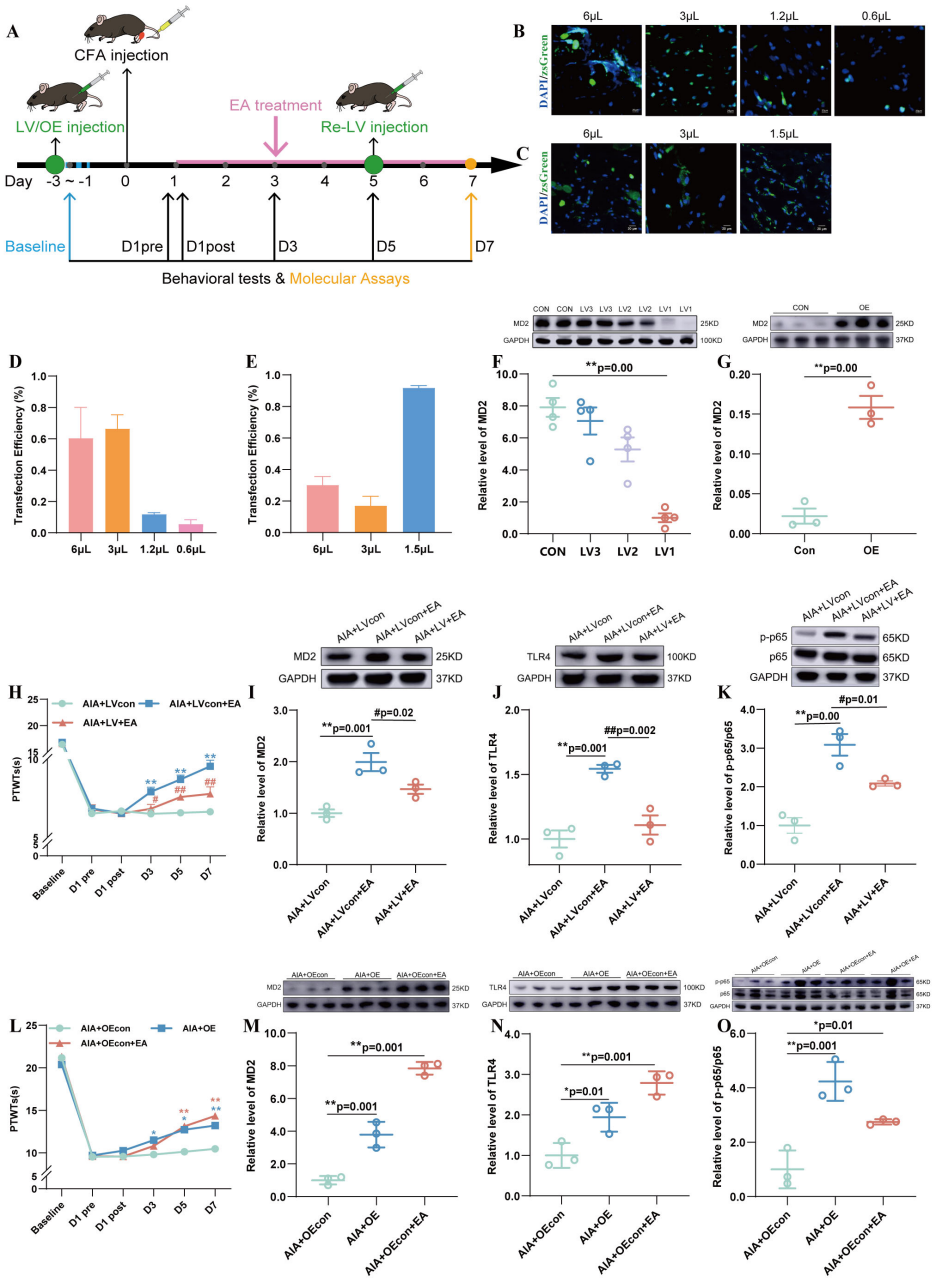
The effects of modulating MD2 in the ST36 acupoint on EA-induced analgesia and on the protein levels of MD2, TLR4, and p-p65/p65. **(A)** Lentivirus injection, behavioral testing, and acupoint stimulation experimental schedule. **(B)** Detection of lentivirus transduction in the ST36 acupoint by IF assay after MD2 knockdown lentivirus injection (n=3). **(C)** IF assay detection of lentivirus transduction in ST36 acupoint after overexpression lentivirus injection (n=3). **(D)** The transfection efficiency of the control lentivirus (FR-376) in ST36 acupoint (n=3). **(E)** The transfection efficiency of the control lentivirus (FV-115) in ST36 acupoint (n=3). **(F)** The expression of MD2 after injection of three different target knockdown lentiviruses were detected by WB (n=4). **(G)** The expression of MD2 after injection of overexpression was detected by WB (n=3). **(H)** The PTWTs were detected on D-3 to D-1, D1pre, D1post to D7 (every other day) (n = 6), ^**^P < 0.01 vs. the AIA+LVcon, ^#^*P* < 0.05 vs. the AIA+LVcon+EA, ^##^*P* < 0.01 vs. the AIA+LVcon+EA. **(I–K)** The expression level of MD2 **(I)**, TLR4 **(J)**, and p-p65/p65 **(K)** (n = 3), ^**^P < 0.01 vs. the AIA+LVcon, ^#^*P* < 0.05 vs. the AIA+LVcon+EA, ^##^*P* < 0.01 vs. the AIA+LVcon+EA. **(L)** PTWTs were detected on D-3 to D-1, D1pre, D1post to D7 (every other day) (n = 6), ^*^P < 0.05 vs. the AIA+OEcon, ^**^P < 0.01 vs. the AIA+OEcon. **(M–O)** The expression level of MD2 **(M)**, TLR4 **(N)**, and p-p65/p65 **(O)** (n = 3), ^**^P < 0.01 vs. the AIA+OEcon.

**Figure 5:**
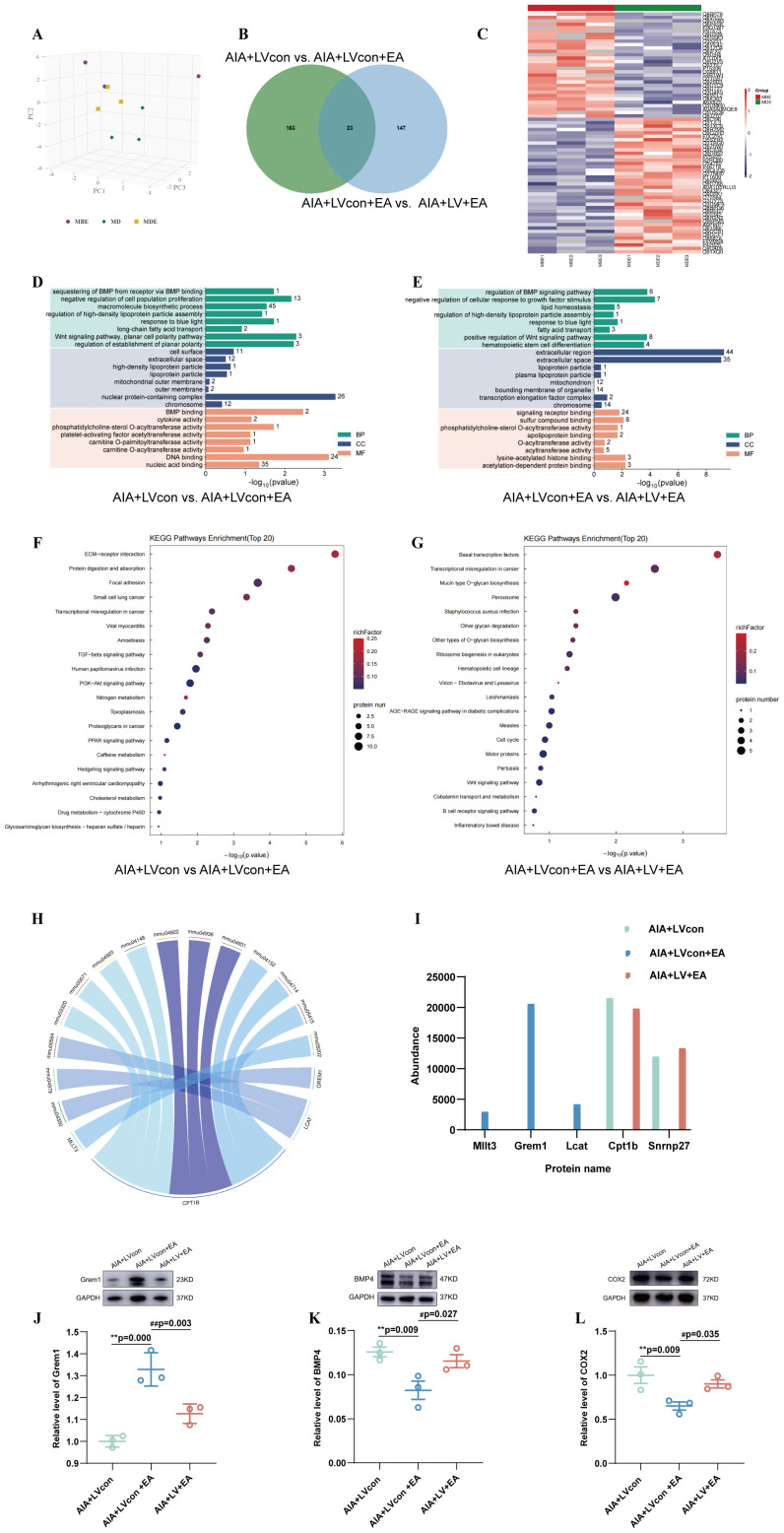
Functional enrichment analysis and distribution of differentially expressed proteins in the spinal cord of mice treated with EA and MD2 knockdown. **(A)** PCA analysis of all samples, each point denotes a sample, and different colors indicate different groups. **(B)** Each color represents a group, and the number of differentially expressed genes is indicated in the figure. **(C)** The hierarchical clustering results are presented as a dendrogram heatmap, with the x-axis and y-axis corresponding to samples and differentially expressed proteins, respectively. In the heatmap, red indicates significant upregulation, blue indicates significant downregulation, and gray represents the absence of quantitative data. **(D, E)** The vertical axis represents the GO level 2 functional annotation information, including molecular function, cellular component, and biological process. The horizontal axis indicates the significance of enrichment, with larger values on the horizontal axis representing a higher significance level of the corresponding GO functions. **(F, G)** KEGG pathway analysis of differential proteins. The vertical axis represents the names of pathways involving differentially expressed proteins. The horizontal axis indicates the significance of enrichment, with larger values signifying a higher level of significance for the enrichment in the corresponding pathway. The color gradient represents the magnitude of the enrichment factor (Rich Factor ≤ 1), with colors closer to red indicating a larger Rich Factor value. **(H)** Circos plot of the relationship between the significantly enriched KEGG pathway and the protein. **(I)** Bar chart showing the expression levels of the 5 key differentially expressed proteins. **(J–L)** The expression level of Grem1 **(J)**, BMP4 **(K)**, COX2 **(L)** (n = 3), ^**^P < 0.01 vs. the AIA+LVcon, ^#^*P* < 0.01 vs. the AIA+LVcon+EA, ^##^*P* < 0.01 vs. the AIA+LVcon+EA.

Membrane was cropped at 35/40 kDa to separate low (<40 kDa: MD2, Grem1 GAPDH) and high molecular weight region (>40 kDa: TLR4, p65, p-p65, TLR4, PKC,BMP4, COX2). Full markers (kDa) are shown on both sides. GAPDH (37 kDa) as a control. The untrimmed full-length membrane is presented in **Figures 1**–**5**-RawData. The GAPDH bands for p65, p-p65, and TLR4 were derived from the same PVDF membrane.

### 2.7 Elisa

The concentrations of IL-1β (EK0394, BOSTER, China) and CX3CL1 (990751.03, R&D SYSTEMS^®^, USA) in the ST36 acupoint were measured using ELISA kits according to the manufacturer’s instructions. After the addition of the stopping solution, the optical density (OD) of each well was determined within 30 minutes using a microplate reader (1712205, BIOTEK, USA) set at 450 nm. The levels of CX3CL1 and IL-1β in the ST36 acupoint were then calculated based on the standard curve and subsequently statistically analyzed.

### 2.8 Co-immunoprecipitation assay

Co-IP assays were performed using an immunoprecipitation kit containing Protein A+G Magnetic Beads (BK0004-02, ACE, China) according to the manufacturer’s protocol. Tissue lysate (1 mg protein) was incubated with either 2 µg of mouse monoclonal anti-TLR4 antibody or 2 µg of mouse IgG isotype control antibody (5415, Cell Signaling Technology, USA) for 1 hour at room temperature. The mixture was then incubated with 20 µL of Protein A/G Magnetic Beads with gentle agitation at 4°C overnight. After centrifugation, the pellets were washed three times with lysis buffer.

The target proteins were detected by WB using antibodies against TLR4 and MD2.

### 2.9 Immunofluorescence staining

The mice were anesthetized by isoflurane inhalation and then subjected to cardiac perfusion with 0.9% saline (20–50 mL) until most of the blood was drained. This was followed by perfusion with 4% paraformaldehyde until the body became completely rigid. Immediately thereafter, the ST36 acupoints were incised, with an incision measuring 5 × 5 mm and a depth of 4–5 mm. The collected tissues were rinsed in phosphate-buffered saline (PBS). Subsequently, these tissues were fixed in 4% paraformaldehyde for 24 hours, then sequentially subjected to gradient ethanol dehydration and xylene clarification. Finally, the tissues were embedded in melted paraffin (P100928-500g, Aladdin, China) for sectioning to obtain cross-sectional samples of the acupoint tissue.

ST36 acupoint sections (7 µm/slide) were deparaffinized, hydrated, and pretreated in a microwave for antigen retrieval. Sections were incubated overnight at 4°C with primary antibodies against MD2, TLR4 (1:5000, sc-293072, Santa, USA), Vimentin (1:200, sc-373717, Santa, USA), Mast Cell Tryptase (1:200, sc-59587, Santa, USA), and F4/80 (1:500, GB113373, Servicebio, China). After washing with PBS, sections were incubated for 1 hour at room temperature with secondary antibodies: HRP-labeled goat anti-mouse IgG (1:500, GB23301, Servicebio, China) and CY3-labeled goat anti-rabbit IgG (1:300, GB21303, Servicebio, China). Nuclei were stained with DAPI reagent (G1012, Servicebio, China) for 10 minutes, followed by washing. Sections were mounted in an anti-fade reagent and examined using a fluorescence microscope (Nikon Eclipse C1, Nikon, Japan). Positive expression of MD2, Vimentin, Mast Cell Tryptase, and F4/80 was indicated by red fluorescence, while TLR4 expression was indicated by green fluorescence. Co-expression of MD2/TLR4, Vimentin/TLR4, Tryptase/TLR4, and F4/80/TLR4 was indicated by combined fluorescence. Staining results were analyzed using ImageJ software (Media Cybernetics, USA).

### 2.10 Lentivirus production and injection

The interfering shRNA target for Ly96 was professionally designed and successfully synthesized by Tianjin Sheweisi Biotech Co., Ltd. Subsequently, the interference vector PLVX-shLy96 was constructed and co-transfected with psPAX2 and pMD2G plasmids into HEK293T cells to package the interference lentivirus. In parallel, PLVX-Puro was co-transfected with psPAX2 and pMD2G to package the control lentivirus. The viral transduction was verified using an IF assay (**Figures 4B, D**). The efficacy of the lentivirus-mediated knockdown was verified (**Figure 4F**), and overexpression was confirmed by WB (**Figure 4G**).

To precisely regulate the local expression levels of MD2 at the ST36 acupoint, multiple injection sites were selected around the acupoint. In the experimental group, lentivirus for knockdown (0.56 µL, FR-1788, 4 × 10^9^ TU/mL) or overexpression (1.5 µL, FC-6312, 5 × 10^8^ TU/mL) was administered 4 days prior to the intervention. In the control groups, the corresponding control lentivirus were administered: knockdown control lentivirus (22.5 µL, FR-376, 1.0 × 10^8^ TU/mL) or overexpression control lentivirus (1.5 µL, FV115, 7.5 × 10^8^ TU/mL). No detectable ZsGreen signal was observed in the anatomical structures adjacent to the ST36 acupoint, systemic organs (liver, spleen), or the brain (**Supplementary Figure S1**).

### 2.11 Proteomic analyses

After homogenization, animal tissue samples were treated with SDT buffer and DTT (40 mM) and mixed at 600 rpm for 1.5 hours at 37°C. After cooling, IAA (20 mM) was added to block reduced cysteine residues, and the samples were incubated for 30 minutes in the dark. Next, the samples were transferred to filters (Microcon units, 10 kDa) and washed with 100 µL of 25 mM NH4HCO3 buffer. Subsequently, trypsin (1:50) was added to the samples and incubated overnight at 37°C. The peptides were desalted on C18 cartridges (Empore™ SPE Cartridges MCX, 30 µm, Waters), concentrated by vacuum centrifugation, and reconstituted in 0.1% formic acid. The peptide content was estimated by UV absorbance at 280 nm. For DIA experiments, iRT calibration peptides were spiked into the samples. The peptides from each sample were analyzed using an Orbitrap™ Astral™ mass spectrometer (Thermo Scientific) connected to a Vanquish Neo system liquid chromatography (Thermo Scientific) in data-independent acquisition (DIA) mode. DIA data were analyzed with DIA-NN 1.8.1.

### 2.12 Statistical analysis

The data are presented as the mean ± SEM. Statistical analysis was performed using IBM SPSS 21.0 software. Multiple measurements at different time points were analyzed by repeated measures analysis of variance (ANOVA), for example PTWTs. The statistics analysis of groups were analyzed by Univariate ANOVA. One-way ANOVA was used for independent samples to compare the differences between groups at each time point. The least significant difference (LSD) test was used if accorded with homogeneity of variance; otherwise, the Dunnett’s T3 method was applied. For data that do not conform to a normal distribution, non-parametric methods were used. Values of *P* < 0.05 or *P* < 0.01 were considered statistically significant (**Figures 3D, H**).

## 3 Results

### 3.1 The analgesic effect of EA on AIA mice model

Prior to the interventions, the PTWTs of each group (the CON, AIA, SHAM and EA groups) were not statistically significant (*P* > 0.05). After the successful establishment of the model, the PTWTs of mice with CFA-induced adjuvant arthritis decreased (*P* < 0.01). After intervention, the PTWTs of the SHAM group (*P* > 0.05) were not significantly different compared with the AIA group. The EA group (*P* < 0.01) increased remarkably compared to the SHAM group on the D3, D5, and D7 (**Figure 1C**).

The levels of spinal cord PKC, CX3CL1 and IL-1β, which are indicators of pain, increased in the AIA group (*P* < 0.01) compared with the CON group. The SHAM group (*P* > 0.05) showed no significant changes compared with the AIA group. In the EA group, PKC and CX3CL1 levels (*P* < 0.01) decreased compared with the SHAM group, while IL-1β levels (*P* > 0.05) remained unchanged (**Figures 1D–F**), the differing responses of CX3CL1 and IL-1β may reflect their distinct temporal dynamics in the inflammatory process or their varying sensitivities to EA intervention.

Before proceeding to molecular validation, we used bioinformatic analysis (STRING database) to predict the MD2-centered interaction network, providing initial biological plausibility for our hypothesis and directing our subsequent focus on key molecules such as TLR4 (**Figure 1G**).

### 3.2 Effect of EA treatment on the expression of MD2, TLR4 and the p-p65/p65 in the ST36 acupoint

Following the confirmation of EA-induced analgesia at ST36 (Section 3.1), we focused on to determine the contribution of MD2 within the acupoint to this effect. To interrogate the underlying mechanism, we performed a series of molecular analyses to assess the expression and functional interaction of key proteins in the hypothesized pathway (**Figure 1**).

The WB assay revealed significant changes in the expression of MD2, TLR4 proteins, and p-p65/p65 in the ST36 acupoint after 7 days of EA treatment. Post-molding, the AIA group (*P* < 0.05, *P* < 0.01) exhibited increased MD2 and TLR4 expression levels, as well as an elevated NF-κB pathway compared to the CON group. After sham EA treatment, there were no significant changes in the expression levels of MD2, TLR4, and p-p65/p65 in the SHAM group compared to the AIA group. Following EA treatment, the expression levels of MD2, TLR4, and the NF-κB pathway in the EA group (*P* < 0.01) were increased compared to those in the SHAM group (**Figures 1I–K**).

Meanwhile, Co-IP assay confirmed the interaction between MD2 and TLR4 in the ST36 acupoint (**Figure 1H**).

### 3.3 Time-course patterns of EA on the expression of MD2, TLR4 in the ST36 acupoint

After establishing the correlation between EA and the upregulation of the MD2 pathway at ST36 (Section 3.2), our aim is to further explore the time-course patterns of MD2 and its downstream molecules in the ST36 acupoint. We measured MD2 expression in the ST36 acupoint on D1post, D3, and D5 to identify when MD2 levels increased in the EA group. The time at which MD2 levels showed a statistically significant elevation in the EA group compared to the SHAM group was identified as the specific time. Subsequently, we tracked TLR4 changes at this specific time (**Figure 2**).

Prior to the interventions, the PTWTs of the CON, AIA, SHAM and EA groups (*P* > 0.05) were not statistically significant. After successful modeling, the PTWTs of AIA, SHAM and EA groups (*P* < 0.01) decreased. After interventions, the PTWTs of the SHAM group (*P *> 0.05) were found to be no difference compared with the AIA group. The EA group (*P* < 0.01) increased remarkably compared to the SHAM group on D3, D5 (**Figures 2B–D**).

The MD2 protein level in the ST36 acupoint of the AIA group showed not significant changes on D1post (*P* > 0.05), but increased on D3 (*P* < 0.01) and D5 (*P* < 0.05) compared to that in the CON group; the SHAM group showed no difference compared to the AIA group on D1post (*P*>0.05), D3 (*P* > 0.05), but increased on D5 (*P* < 0.05); and that in the EA group (*P* < 0.05) increased on D5 compared to that in the SHAM group (**Figures 2E–G**). Therefore, we speculate that MD2 may have undergone a significant increase on D5. Afterwards, we examined the expression of TLR4 in the ST36 acupoint on D5 and found that there was no change (**Figure 2H**).

Based on the presented data, we conclude that the impact of EA on MD2 likely commences on D5, with the activation of the MD2/TLR4/NF-κB pathway possibly occurring on D7 (**Figures 1I–K**).

### 3.4 The cellular localization of MD2 and TLR4 in the ST36 acupoint

After establishing the temporal profile of MD2 and its downstream signaling molecules at the ST36 acupoint (Section 3.3), we next sought to gain further insight by determining their spatial distribution. Therefore, we investigated the cellular localization of the MD2/TLR4 complex within key immune cells at the acupoint to provide an anatomical context for the observed molecular changes (**Figure 3**).

The number of co-stained cells in a fixed visual field (200 × 200 μm) in the ST36 acupoint were analyzed to reflect the co-localization of MD2 and TLR4, TLR4 and the major cells (fibroblast, mast cell, macrophage). After molding, the IF results indicated the co-localization of MD2 and TLR4, TLR4 and Vimentin, TLR4 and Tryptase, TLR4 and F4/80. MD2 and TLR4 co-stained cells in the AIA group (*P* < 0.05) increased compared to that in the CON group. After EA treatment, the number of co-stained cells in the EA group (*P* < 0.01) increased compared to the AIA group (**Figures 3A, E**). TLR4 and Vimentin co-stained cells in the AIA group (*P* < 0.01) increased compared to CON group, the EA group (*P* < 0.01) increased when compared to the AIA group (**Figures 3B, F**). TLR4 and Tryptase co-stained cells in the AIA group increased compared to CON group but there was no statistical significance (*P* > 0.05), and the EA group (*P* < 0.05) increased when compared to the AIA group. Still, TLR4 with F4/80 co-stained cells in the AIA group (*P* > 0.05) increased compared to the CON group, the EA group (*P* > 0.05) increased compared to the AIA group, but there was no statistical significance (**Figures 3D, H**).

### 3.5 Effects of MD2 regulation in the ST36 acupoint on EA-induced analgesia, TLR4 and p-p65/p65 expression

Based on the aforementioned research findings, we examined the effects of MD2 knockdown or overexpression on mechanisms underlying EA-induced analgesia and on the levels of MD2, TLR4, and p-p65/p65 to investigate its exact biological role in pain modulation at the ST36 acupoint (**Figure 4**).

FR-376 was used as the control lentiviruses for the MD2 knockdown group, while FV115 was used as the control lentiviruses for the MD2 overexpression group. To ensure more precise knockdown of MD2, we designed three lentiviruses targeting different sites (LV1: FR-1788, LV-mly96-shRNA1, LV2: FR-1789, LV-mly96-shRNA2, LV3: FR-1790, LV-mly96-shRNA3) and used WB assay to identify the knockdown status of these lentiviruses in the ST36 acupoint (**Figure 4F**). First, IF analysis revealed that the optimal concentration for the knockdown lentivirus was 22.5 × 10^5^ TU/µL (**Figures 4B, D**), while the optimal concentration for the overexpression lentivirus was 5 × 10^5^ TU/µL (**Figures 4C, E**). Second, WB results showed that LV1 reduced the expression level of MD2 protein (**Figure 4F**), while the overexpression lentivirus (OE) increased the expression level of MD2 protein (**Figure 4G**). Another experiment demonstrated that the volume disparity itself, under our controlled conditions, did not elicit substantially different local tissue reactions (**Supplementary Figure S2**).

The PTWTs values of the AIA+LVcon+EA group (*P* < 0.01) increased compared to that in the AIA+LVcon group on D3, D5, and D7. After MD2 was knockdown, the PTWTs in the AIA+LV+EA group (*P* < 0.05, *P* < 0.01) decreased on D3, D5, and D7 compared with the AIA+LVcon+EA group (**Figure 4H**). Moreover, the levels of MD2, TLR4, and p-p65/p65 in the AIA+LVcon+EA group (*P* < 0.01) elevated compared with that in the AIA+LVcon group. The levels of MD2, TLR4 and p-p65/p65 in the AIA+LV+EA Group (*P* < 0.05, *P* < 0.01) were decreased compared to those in the AIA+LVcon+EA group (**Figures 4I–K**).

After MD2 overexpression, the PTWTs in the AIA+OE group (*P* < 0.05, *P* < 0.01) increased on D3, D5, D7 compared with the AIA+OEcon. The PTWTs in the AIA+OEcon+EA group (*P* < 0.01) elevated compared with the AIA+OEcon group on D5 and D7 (**Figure 4L**). In addition, the levels of MD2, TLR4, and p-p65/p65 in the AIA+OE group (*P* < 0.05, *P* < 0.01) increased compared with the AIA+OEcon. The levels of MD2, TLR4, and p-p65/p65 in the AIA+OEcon+EA group (*P* < 0.05, *P* < 0.01) increased compared with that in the AIA+OEcon group (**Figures 4M–O**).

In summary, these results indicated that modulation MD2 in the ST36 acupoint can influence the analgesic effects of EA treatment, as well as the expression of TLR4 and p-p65/p65.

### 3.6 Proteomics reveals the impact of MD2 regulation in the ST36 acupoint on the spinal analgesic pathway

Our findings (Section 3.5) confirmed that the MD2/TLR4/NF-κB pathway in the ST36 acupoint was a key local mechanism mediating EA analgesia. However, how this local signal influences the central nervous system to produce analgesic effects remained unclear. To address this, we performed a proteomic analysis of the spinal cord in mice with MD2 knockdown in the acupoint area, aiming to identify EA-regulated proteins at the spinal level that are dependent on acupoint MD2 (**Figure 5**).

The results of PCA assay revealed high sample dispersion between groups but high sample aggregation within groups (**Figure 5A**).

The clear segregation and clustering of the data indicated that distinct proteins existed between groups. Then the folder change threshold of 1.5 and *P* < 0.05 was selected to filter differential proteins. The numbers of differential proteins were 163 between the AIA+LVcon group and AIA+LVcon+EA group, 147 between the AIA+LVcon+EA group and AIA+LV+EA group, and the differential proteins intersection of these three groups was 23 (**Figure 5B**).

Furthermore, to isolate the subset of spinal cord proteins whose EA-induced regulation depends on MD2, we compared the proteomic profiles of the AIA+LVcon, AIA+LVcon+EA, and AIA+LV+EA groups. This comparison revealed significant alterations in the expression profiles of multiple proteins. Heatmap of differential proteins were shown all up-regulated and down-regulated proteins in all conditions and the significantly differentially expressed proteins obtained can effectively separate the comparison groups (**Figure 5C**).

Application of GO Analysis for the functional classification of differentially expressed proteins in the spinal cords, the most significant data were included to do histogram analysis. The differential proteins regulated by EA treatment in the AIA+LVcon+EA group are primarily enriched in cellular components and molecular functions, the most abundant cellular component was extracellular region, extracellular space, etc.; the most concentrated molecular function was signaling receptor binding, sulfur compound binding, etc. compared to those in the AIA+LVcon group (**Figure 5D**). In the AIA+LV+EA group, compared to the AIA+LVcon+EA group, the differential proteins regulated by MD2 are primarily enriched in biological process and molecular functions, the most enriched biological process was negative regulation of cell population proliferation, Wnt signaling pathway, planar cell polarity pathway, regulation of establishment of planar polarity, etc.; the most concentrated molecular function was BMP binding, phosphatidylcholine-sterol O-acyltransferase activity, DNA binding, etc. (**Figure 5E**).

To further investigate the enriched pathway of these differential proteins, the KEGG pathway analysis was adopted. In the analysis of the top 20 differentially regulated pathways across the AIA+LVcon, AIA+LVcon+EA, and AIA+LV+EA groups, the most significant pathways as for p-value were human papillomavirus infection, the PI3K-Akt signaling pathway, the PPAR signaling pathway, etc. The most protein enriched pathways were ECM-receptor interaction, focal adhesion, the PI3K-Akt signaling pathway, and the TGF-β signaling pathway, etc. (**Figures 5F, G**).

To elucidate the signal pathways associated with the effects of EA in the spinal cord following MD2 knockdown within the ST36 acupoint, differentially expressed proteins in the CON, AIA, SHAM, and EA groups were analyzed using KEGG pathway enrichment. We focused on inflammation pathways in the spinal cord that are pertinent to pain processing and showed the detailed relationships between them (FC>1.5) using a Circos graph (**Figure 5H**).

Five key proteins (Mllt3, Grem1, Lcat, Cpt1b, and Snrnp27) were identified through the KEGG database in the relevant pathways (**Figure 5I**). We found that the differentially expressed proteins related to the TGF-β pathway in the spinal cord, such as Grem1, are closely related to the changes in MD2 at the acupoint area. The Grem1 showed an obvious opposite trend in the AIA+LVcon group and the AIA+LVcon+EA group. Based on GO Pathway analysis and previous studies (16), Grem1 may inhibit the expression of BMP4, thereby suppressing the inflammatory response in the spinal cord and contributing to the development of spinal pain. We subsequently examined the protein expression of Grem1 and BMP4 using WB.

The levels of Grem1 in the AIA+LVcon+EA group (*P* < 0.01) elevated compared with that in the AIA+LVcon group. The mice in the AIA+LV+EA group (*P* < 0.01) decreased when compared to that in the AIA+LVcon+EA group (**Figure 5J**). The levels of BMP4 and COX2 in the AIA+LVcon+EA group (*P* < 0.01) decreased compared with that in the AIA+LVcon group. The mice in the AIA+LV+EA group (*P* < 0.05) increased when compared to that in the AIA+LVcon+EA group (**Figures 5K, L**).

## 4 Discussion

In this experiment, we focused on the role of MD2 in the ST36 acupoint in the analgesic mechanism of EA. We found that the analgesic effect of EA emerged on D1post and stabilized by D3 (**Figures 1C**, **3B–D**). The activation of the TLR4/NF-κB pathway by MD2 occurred on Day 7 of EA treatment (**Figures 1I–K**). Based on this, we further examined the expression changes of MD2 in the ST36 acupoint on D1post, D2 (**Supplementary Figure S3**), D3, and D5, and found that the level of MD2 increased on D5 (**Figures 2E–G**). Meanwhile, we detected the expression levels of TLR4 and found that its expression remained unchanged after 5 days of EA treatment (**Figure 2H**), but increased after 7 days of EA treatment (**Figure 1J**). Subsequently, the expression levels of p-p65/p65 also increased after 7 days of EA treatment (**Figure 1K**).

To establish the functional necessity of MD2, we regulated the expression of MD2 in the ST36 acupoint using lentivirus. Results showed that MD2 knockdown partially reversed EA-induced analgesia (**Figure 4H**) and suppressed local expression of MD2, TLR4, and p-p65/p65 at the acupoint (**Figures 4I–K**). In contrast, overexpression of MD2 partially mimicked the analgesic effect of EA (**Figure 4L**) and upregulated these pathway components (**Figures 4M–O**). These functional experiments confirm that MD2 is a necessary factor for the full development and maintenance of EA analgesia.

Integrating the chronological and functional data, we propose that within the ST36 acupoint microenvironment, MD2 serves as a pivotal mediator in EA-induced analgesic signaling. Through its mediated cascade, sustained EA stimulation generates cumulative effects that progressively activate innate immune signaling, thereby synergistically promoting the establishment and maintenance of analgesia. However, current evidence indicates that MD2 contributes partially to this process and does not represent the exclusive mechanism.

Among the proteins bidirectionally regulated by EA and MD2 knockdown, Grem1 was prioritized due to its consistent expression pattern (upregulated by EA and reversed by MD2 knockdown) and its established role in spinal nociceptive processing (21). KEGG database analysis indicated that Grem1 functions as an antagonist of BMP in the TGF-β signaling pathway, while GO analysis consistently revealed that the differentially expressed proteins were highly enriched in the regulation of BMP signaling receptor activity (BMP receptor sequestration), a process associated with inflammatory pain. As a secreted antagonist of the BMP signaling pathway, Grem1 competitively inhibits BMP4, which promotes microglial activation and central sensitization (22, 23), thereby attenuating spinal inflammation by suppressing the expression of COX2, its a key mediator of inflammatory pain (24, 25). This coherent Grem1-BMP4-COX2 axis is well-supported in pain research and provides a biologically plausible mechanism for MD2-mediated EA analgesia. Western blot analysis further confirmed that Grem1 suppresses the expression of BMP4 and COX2, supporting the functional relevance of this pathway. Compared to Grem1, other candidate proteins (e.g., CPT1B) have a more indirect connection to pain, primarily influencing metabolic pathways rather than nociceptive signaling. Additionally, proteins like Tmpo, Sumf2, Flt3, and Tbck, though affected by EA, lack sufficient evidence linking them to inflammatory pain.

Although the present study has delineated the spinal GREM1/BMP4 signaling pathway in response to EA analgesia mediated by acupoint MD2, our proteomics analysis also revealed a number of potential proteins within the spinal cord that may participate in EA-mediated pain relief. For instance, Stat2 and Flt3 are key signaling molecules in the interferon and microglial homeostasis pathways, respectively, highlighting EA’s potential to modulate neuroimmune responses in the spinal dorsal horn (26, 27). Meanwhile, Caveolin-1 (Cav1), a critical regulator of membrane receptor signaling (28), and Nestin (Nes) (29), a marker associated with neural plasticity, suggest additional mechanisms through which EA may stabilize neuronal excitability and promote adaptive rewiring within pain-related neural circuits. The potential roles of these candidate proteins collectively underscore that EA analgesia is a multifaceted process likely involving intricate neuro-immune crosstalk and neuronal plasticity. A deeper understanding of how these proteins participate in the pain management process and how EA intervenes in these pathways will not only enrich our fundamental knowledge of pain biology but also pave the way for developing novel targeted therapeutic strategies that mimic or enhance the beneficial effects of acupuncture.

Although the analgesic effect of EA is initiated locally through the MD2/TLR4/NF-κB pathway at the ST36 acupoint, its expression depends on integrative processing within the central nervous system. While the precise signaling mechanism linking acupoint stimulation to spinal cord responses remains incompletely elucidated, our proteomic analysis confirms that localized manipulation at ST36 is capable of eliciting specific molecular alterations in the spinal cord. This evidence supports the existence of acupoint-spinal cord communication. While a functional link is established, the intermediary transmission mechanisms remain unclear. Based on existing literature, we propose two plausible pathways: (1) Neural transmission: sensory nerve endings densely distributed around the ST36 acupoint may transmit signals triggered by MD2 pathway activation to the spinal cord, thereby modulating gene expression in dorsal horn neurons (30, 31). (2) Humoral factors: changes in the local immune microenvironment at the acupoint may lead to the release of certain cytokines or neuropeptides into the circulation, which could then act remotely on the spinal cord (31). Future studies employing selective nerve blockade, local denervation of the acupoint, or systemic measurements of humoral factors will be essential to precisely delineate the communication mechanisms of this “acupoint-spinal cord axis.

The partial reversal of EA analgesia by MD2 knockdown, and the incomplete recapitulation by its overexpression, can be attributed to two non-mutually exclusive factors. Firstly, from a technical perspective, the efficiency of lentivirus-mediated gene delivery in vivo may not achieve complete (100%) knockdown or overexpression in all target cells at the acupoint site, which could partly account for the observed partial effects. More importantly, and from a mechanistic standpoint, the concept that EA analgesia involves a complex interplay of mechanisms is well-supported. At the acupoint microenvironment, EA simultaneously modulates other pattern recognition receptors (e.g., TLR2 or the NLRP3 inflammasome) and distinct neuro-immune pathways, potentially involving the regulation of neuropeptides such as substance P or corticotropin-releasing factor. This multi-target mode of action is consistent with the holistic philosophy of acupuncture. Consequently, our study positions the MD2-TLR4 pathway as a significant, but not solitary, contributor within a broader analgesic network.

This study revealed that EA stimulation at the ST36 acupoint concurrently enhanced MD2/TLR4 signaling activity in macrophages, fibroblasts, and mast cells within the acupoint microenvironment. A key, unresolved question is which of these cell types plays the dominant role in initiating EA-induced analgesia. However, our data more support the possibility that the effect of EA is not dependent on a single cell type but rather stems from a highly coordinated network of intercellular communication. Based on existing literature, fibroblasts, as the primary stromal cells in connective tissue where the acupoint is located, may act as the “primary sensors” of mechanical stimulation (needling), releasing initial signaling factors to activate and recruit immune cells (32). Macrophages, leveraging their potent immunomodulatory capabilities, may then function as “signal amplifiers and integrators,” playing a central role in amplifying and sustaining the inflammatory/anti-inflammatory balance (33). Furthermore, mast cells, which are densely distributed at acupoints and whose involvement in acupuncture effects is well-documented (34, 35), may contribute through rapid responses and by regulating local blood flow within the neuro-immune regulatory network of the local microenvironment. This suggests that the EA effect is more likely to emerge from a multi-cellular synergistic network response rather than a linear pathway driven by an individual cell type. Consequently, the acupoint may function akin to a “biological signal processor,” where distinct cell types, each performing specialized roles, collectively transduce the physical stimulus (needling) into a complex biochemical signal.

The selection of stimulation frequency is critical for EA-induced analgesia. Previous studies. The selection of stimulation frequency is critical for EA efficacy. Low-frequency EA (e.g., 2 Hz) is preferred for chronic deep-tissue pain due to its systemic, long-lasting analgesia mediated by Aδ/C fibers and neuropeptide release via the HPA axis (36). In contrast, high-frequency EA (e.g., 100 Hz) acts rapidly but segmentally, suited for acute superficial pain via Aβ fiber-mediated spinal inhibition (37). To integrate these benefits, we employed a 2/15 Hz alternating paradigm aimed at sustaining analgesia while reducing tolerance. In addition, the selection of experimental subjects is equally important. This study used only male mice to control for confounding hormonal fluctuations across the estrous cycle, thereby strengthening internal validity despite limiting generalizability. Future work in females is needed to test the sex-specificity of the MD2/TLR4 mechanisms identified here.

## 5 Conclusions

This study elucidates a bidirectional regulatory mechanism underlying EA-induced analgesia mediated through the ST36 acupoint-localized inflammation-MD2 axis. The inflammatory microenvironment at the acupoint upregulates MD2 expression, specifically activating the TLR4/NF-κB signaling cascade in fibroblasts and mast cells, which subsequently modulates the dynamic equilibrium of the Grem1/BMP4/COX2 axis in the spinal dorsal horn. Notably, EA amplifies the MD2-mediated “inflammatory priming” effect at the acupoint, achieving synchronized regulation of localized inflammatory status and targeted modification of spinal nociceptive pathways. These findings not only confirm MD2 as a critical molecular hub bridging acupoint microenvironment with central neuromodulation, but also advance the theoretical framework for acupoint therapy by clarifying the upstream NF-κB pathways activation mechanism and establishing the “local-systemic” synergistic principle.

## Data Availability

The data presented in the study are deposited in the Zenodo repository, accession number 10.5281/zenodo.17796434.
