# Supplementary figures and images for: Electroacupuncture alleviates pain by activating the MD2/TLR4/NF-κB pathway in the ST36 acupoint
Source: Front Immunol. 2026 Jan 29;16:1626755. doi: 10.3389/fimmu.2025.1626755 (PMC12893965; doi:10.3389/fimmu.2025.1626755)

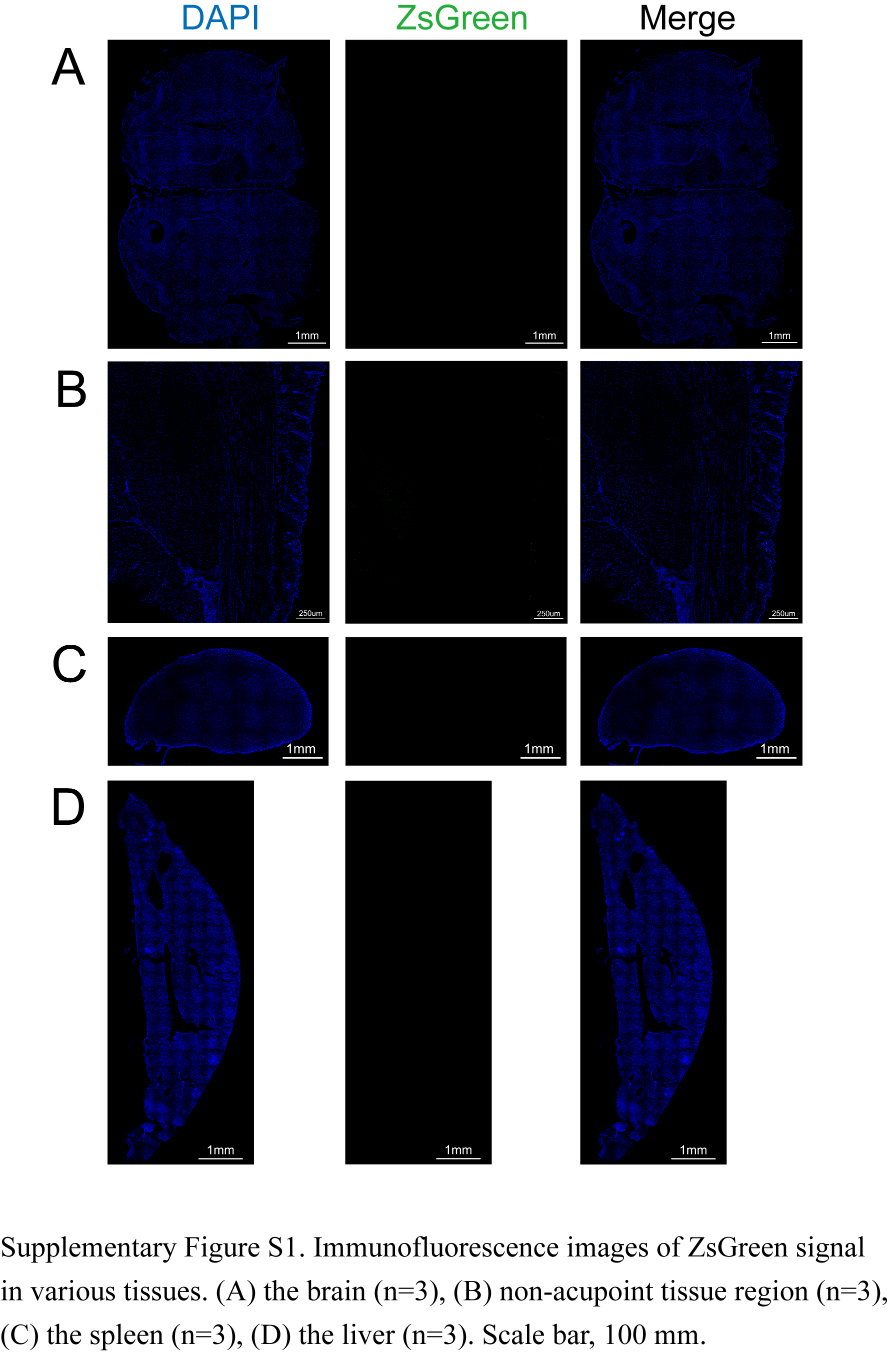

Supplement: Supplementary file 1 [file Image1.tif]

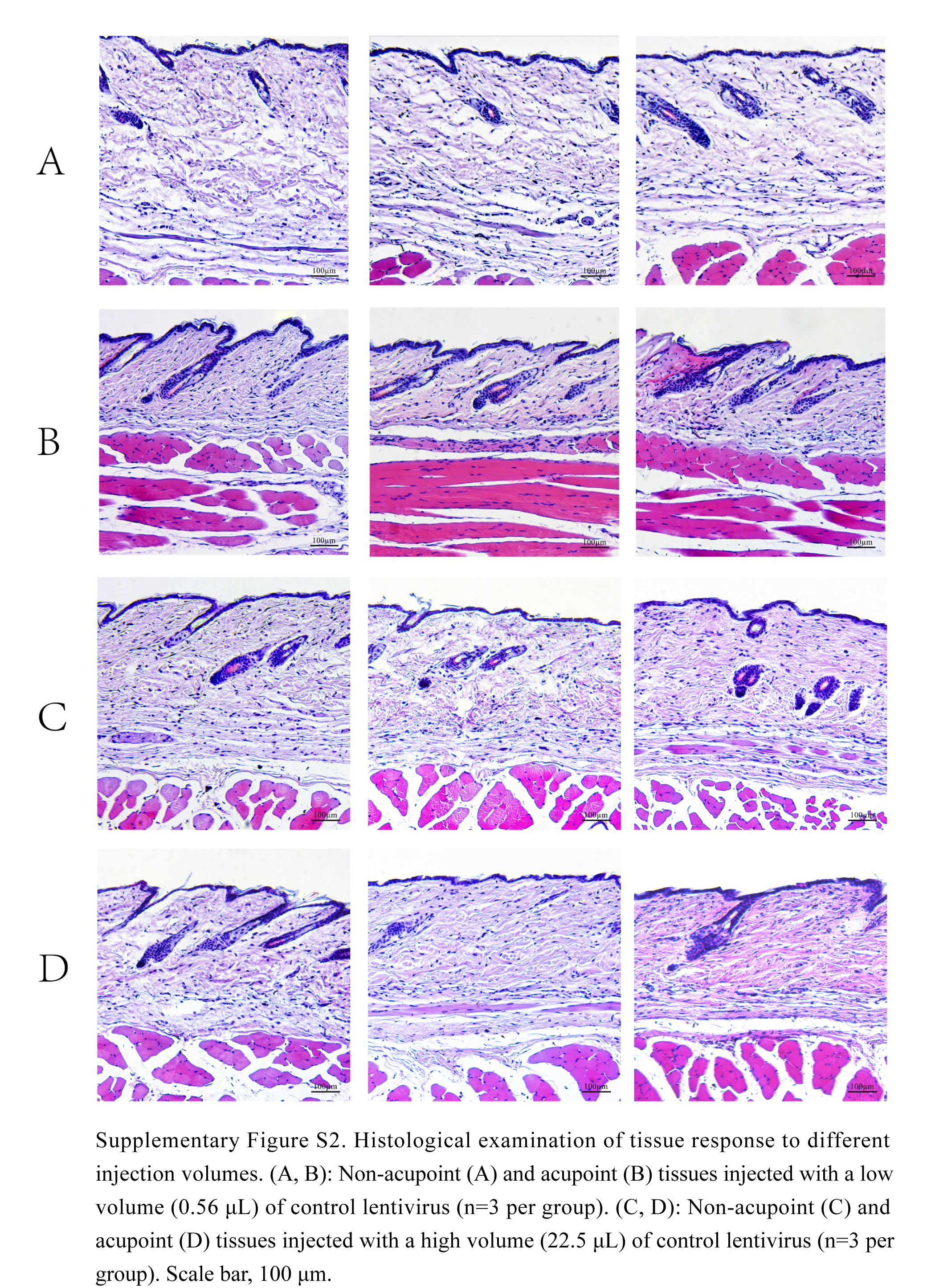

Supplement: Supplementary file 2 [file Image2.tif]

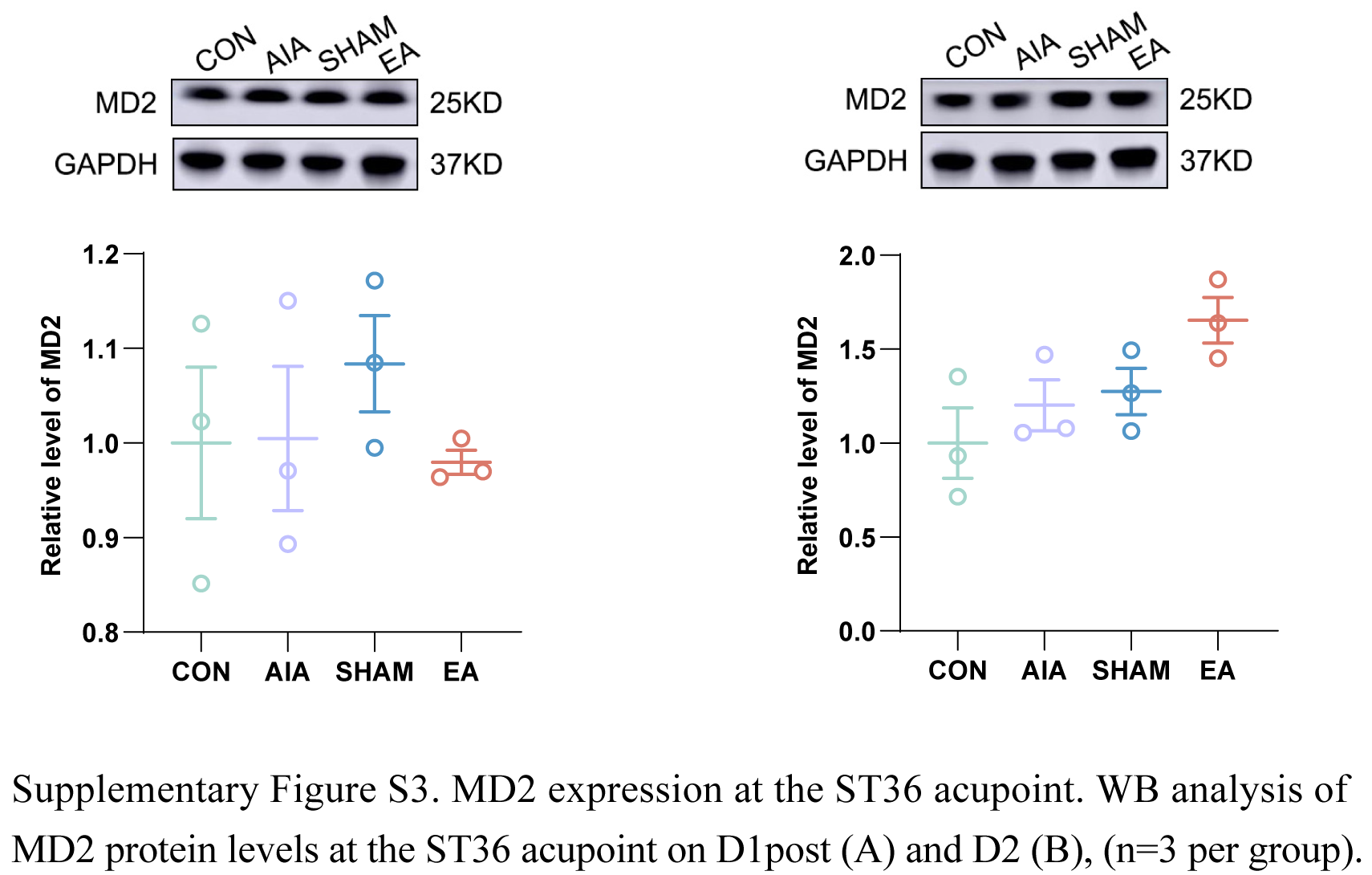

Supplement: Supplementary file 3 [file Image3.tif]

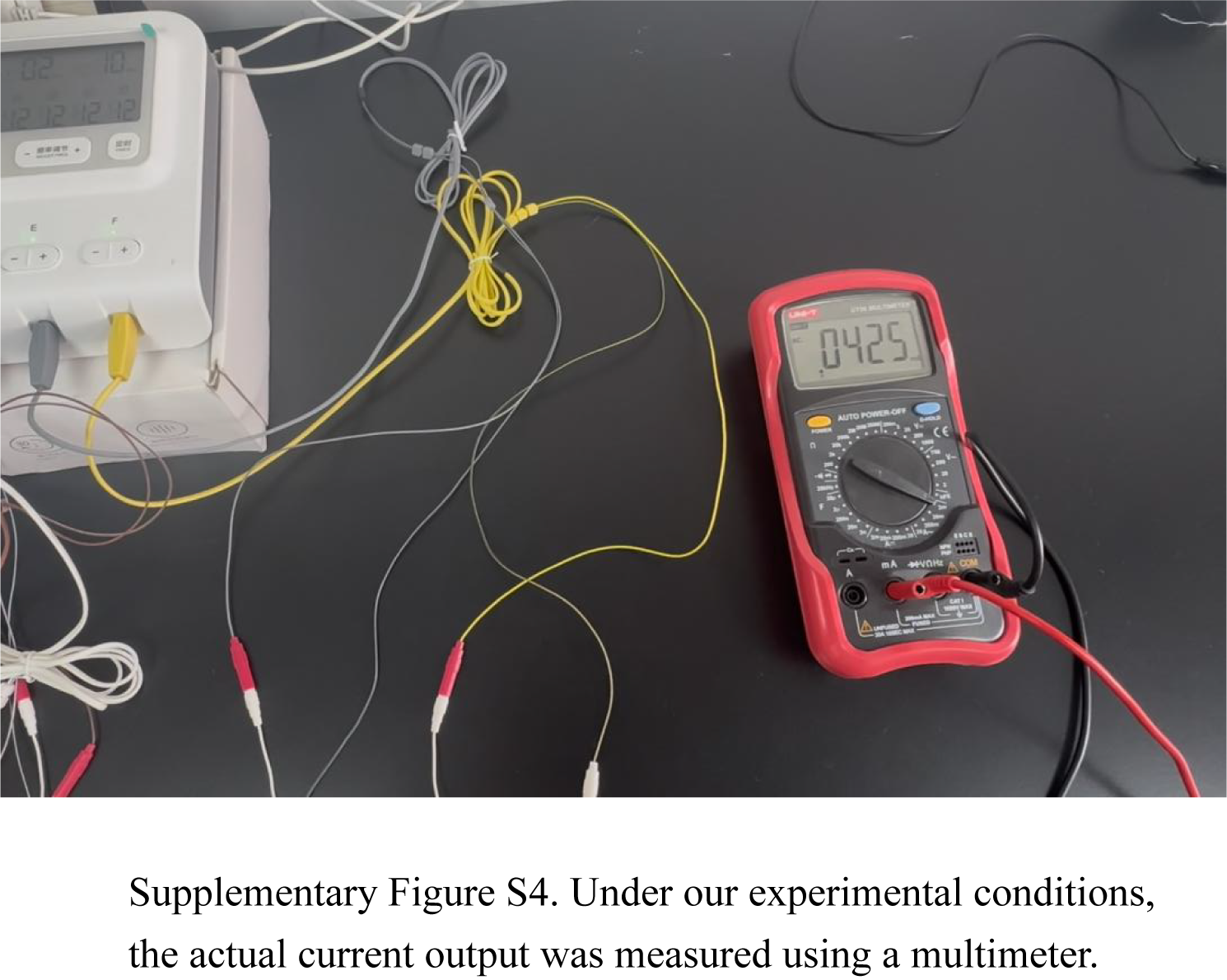

Supplement: Supplementary file 4 [file Image4.tif]
